# The Fibre Requirements of Horses and the Consequences and Causes of Failure to Meet Them

**DOI:** 10.3390/ani13081414

**Published:** 2023-04-20

**Authors:** Colette Ermers, Nerida McGilchrist, Kate Fenner, Bethany Wilson, Paul McGreevy

**Affiliations:** 1 School of Environment and Rural Science, University of New England, Armidale, NSW 2351, Australia; 2 Equilize Horse Nutrition Pty Ltd., Tamworth, NSW 2340, Australia; 3 School of Agriculture and Food Science, The University of Queensland, Gatton, QLD 4343, Australia; 4 School of Life and Environmental Science, The University of Sydney, Sydney, NSW 2006, Australia

**Keywords:** nutrition, fibre, welfare, behaviour, gastric ulcers, hindgut, microbiome

## Abstract

**Simple Summary:**

After water, fibre is the most important component of a horse’s diet. Its value in providing energy is too often underestimated. The energy availability and protein content of pastures and preserved forages depend on the pasture species and the maturity of the pasture at either grazing or cutting. Foraging is a core natural behaviour of horses and ponies and, when possible, takes up most of their daily time budget. Insufficient foraging opportunities result in behaviours that either mimic foraging behaviour or represent an oral occupation. Many of these behaviours are related more to the foraging activity than to the fibre intake. Even those animals on concentrated rations that are rich in fibre are associated with behavioural activities related to a lack of foraging opportunity. Even though foraging directly affects fibre intake, most studies have focused on the behavioural activity of foraging and associated chewing. Replacing starch in a high-energy diet with a fibrous alternative greatly reduces the risk of gastrointestinal disease and improves digestion, body condition, behaviour, immune function, athletic performance, and adaptation to weaning. In many cases, failing to feed the horse its fibre requirement reflects a lack of knowledge on the part of the owner.

**Abstract:**

Failure to meet the minimum forage requirement of 1.5% of the horse’s bodyweight and the opportunity for foraging for a minimum of 8 h a day (not going without this opportunity longer than four to five consecutive hours) can have both physiological and behavioural consequences. To provide an energy source for horses, rations often include starch rather than fibre. This can result in health issues related to the gastrointestinal tract (GIT) in the horse. In the stomach, the main concern is equine gastric ulcer syndrome (EGUS) and, more specifically, equine squamous gastric disease (ESGD). Ulcerations are caused either by increasing acidity in the stomach (from starch ingestion and reduced saliva production) or splashing of acidic juices caused by a lack of a forage barrier prior to exercise or prolonged periods without fibrous feed intake, which allows the stomach to collapse and spread acidic gastric fluids into the upper squamous regions of the stomach. In the hindgut, starch that has escaped digestion in the small intestine causes microbial instability and increased production of volatile fatty acids (VFA) and lactic acid. This puts horses at great risk for acidosis and subsequent laminitis. Shifts in the hindgut microbiota will also affect a horse’s behaviour via the gut-brain axis, as well as potentially compromise immune function. Reduced fluid intake caused by reduced saliva production can result in colic. Choosing a fibrous alternative for starch in a high-energy diet greatly reduces the risk of EGUS and acidosis and improves digestion, GIT pH, body condition, behaviour, immune functions, and performance. Providing hay can reduce crib-biting, wood-chewing, coprophagia, the consumption of bedding, aggression, and stress, and subsequently increase social bonding and affiliation with conspecifics. Adequate fibre intake is related to reduced clinical signs of EGUS, reduced reactivity, and better adaptation to weaning. Lignophagia (wood chewing) has also been observed in horses that are foraging, and this is thought to reflect low fibre content in the available forage (for example, early vegetative, lush pasture).

## 1. Introduction

The equine gastrointestinal tract has evolved to process large amounts of fibre, as one would expect of any grazing and browsing herbivore. Free-ranging horses can survive on pasture and other forages they browse without all the high-starch concentrates that are commonly fed to domestic horses. In developing diets for domestic horses, owners or trainers often focus too much on the high-starch concentrates they feed, while paying too little attention to the forage components of a diet [1,2]. It is not clear what factors lead to this erroneous focus, but the role of commercial feed manufacturers merits consideration. They often consider forages as a source of insufficient energy for the horses’ activities and so replace much of the fibre with high-starch diets [3,4]. Studies have shown that horses can undertake intense activities such as harness racing while on a forage-only diet [5]. Horses on forage-only diets have much greater microbial diversity in the hindgut and better microbial stability [4,6] than those on high-starch diets. So, for many stabled horses, especially those that rely exclusively on their carers to provide a balanced, nutritious diet, an improved focus on the fibre content of the ration has merit. Most importantly, the correct fibre content in a ration that is presented in an equi-centric manner and in a palatable form [7] can optimise animal welfare [8]. This review considers how the fibre requirement of a domestic species is determined and what the current recommendations are for fibre intake in horses. It then explores the short-term and long-term behavioural and physiological consequences of feeding horses less than the minimum daily requirement of fibre.

## 2. Understanding Fibre Requirements

### 2.1. What Is Fibre?

Plant fibres exist as various forms of carbohydrates; hence, they are also known as structural carbohydrates, including lignin, cellulose, and hemicellulose [9]. They can be found in many different commercial feedstuffs. In nature, fibre is found mostly in the stem rather than the leaves of grasses and shrubs [10]. In preserved feed, fibre is found in forages or roughages [8].

Forage (as a noun) is defined as preserved cuttings of plants, such as hay, haylage, and silage [11]. Meanwhile, so-called roughage is a term that covers coarse forage such as straw with large proportions of indigestible fibres. Fibre is also found in a highly digestible form in by-product ingredients such as legume hulls or sugar beetroot pulp. The voluntary intake of fibre, either on pasture or fed by a caretaker, is commonly known as foraging [8]. As fibre is concentrated in the stem of grass, fibre content in pasture increases with the sward’s maturity. So, forages cut at different stages of maturity will also differ in fibre content. As a pasture sward matures, it transitions from the vegetative phase to the reproductive phase. In the reproductive phase, there is a higher stem-to-leaf ratio, raising the relative fibre content of the dry matter. An increase in fibre content comes with a decrease in digestibility and in the protein content of the plant. Forage that has been cut from pasture in a vegetative state is higher in energy and protein content and lower in fibre than forage cut from a mature sward, but to determine its full nutritional value as a nutrient for horses, a full nutritional analysis is needed [12]. Even hay cut from the same paddock can differ in nutritional value [8], especially if cut at different times of the year [13]. Routine feed analyses classify fibre in feedstuffs as acid detergent fibre (ADF) or neutral detergent fibre (NDF). The amount of NDF is directly related to the amount of fibre horses can consume in a day.

### 2.2. Fibre Digestion in Horses

In horses, fibres are gathered by the muzzle and incisors before being chewed by the premolars and molars approx. 3400 times per kg of hay [14] or 43,000 times a day when fed hay ad libitum [15] before being swallowed. In the stomach, fibres are exposed to hydrochloric acid and the digestive enzymes lipase and pepsin [16] before being digested via microbial fermentation in the caecum and colon of the hindgut [17]. Fibres are broken down by the bacteria, fungi, and protozoa living in the hindgut. Digestion of fibres in the hindgut results in the production of volatile fatty acids (VFA), notably acetate, butyrate, and propionate [6]. These are absorbed and used as direct energy sources by the horse. The amount of energy gained from fibre varies with different quality fibres and depends on their readily degradable fibre content. Pasture and forages with large amounts of readily degradable fibre are excellent sources of energy for horses [6]. Other by-products of the fermentation of fibre include essential amino acids and vitamins such as the B-group vitamins, including biotin [9,18,19].

The flora responsible for fibre digestion in the hindgut require acidity levels in the hindgut to remain close to neutral and should not be allowed to descend below pH 6.2 for extended periods [20] to be most productive for fermentation. At a pH of less than 6.2, concentrations of beneficial fibre-fermenting bacteria will begin to decline. The delivery of starch to the hindgut will cause a lowering of the hindgut pH, and a lack of fibre quantity or diversity can cause hindgut microbiome disturbances. The absence of fibre or the presence of starch are the major factors causing hindgut instability [21], which can then result in reduced fibre fermentation [22].

### 2.3. Determining Fibre Requirements

When determining the nutritional requirements of animals, it is most common to build diets around the basic need for energy. In herbivores, fibre is used to provide a major portion of dietary energy, and therefore fibre requirements are often linked to energy requirements. However, in horses, the minimum requirement of fibre relates more to time spent foraging and the need to maintain fibrous gut fill in the stomach and hindgut than the nutritional value of the fibre or the energy obtained [8].

In nature, horses forage for the majority of their time in a 24 h period [23,24,25]. Time spent foraging is affected by the time of year. Predator presence and disturbance by flies and mosquitos will affect foraging time, as will weather patterns. Foraging is only stopped voluntarily for a maximum of 3 h at a time [26].

To mimic the free-ranging state in order to minimise behavioural changes and disease states, domestic horse-keepers need to provide horses with access to forages at all times. When horses are denied ad libitum forage, they may display altered behaviour and redirect foraging to another target substrate [26]. For example, stabled horses might eat their bedding and, sometimes, faecal material [8]. Furthermore, diet composition can affect behaviour [27].

More than six hours of no access to forage has been found to significantly increase the risk of gastric ulcers in stabled horses [28]. Both the fibre and the saliva created in chewing them are important buffers that are used by the horse to maintain normal gastric pH, health, and physiology. When low-fibre diets are fed, the acidity in the stomach is increased, which in turn increases the risk of Equine Squamous Gastric Disease (ESGD) [29,30]. Further, low-fibre diets may increase the risk of colic due to low fibre in the hindgut [31]. Therefore, when considering the fibre requirement in horses, the time spent foraging is of great importance, as is the amount of gut fill, saliva production, and buffering the fibre will provide.

In some herbivores, the amount of fibre provided is based on the animals’ requirement for energy. However, in horses, it is a case of adjusting the quality of fibre in the diet in order to control energy intake without reducing the bulk or weight of fibre provided in the diet. As an example, ponies are often on a restricted-energy diet. If they were fed a high-quality, high-energy forage, the amount of fibre fed would be less than is required to provide adequate foraging time and for optimum gut fill and saliva production. If they are instead fed low-quality, low-energy forages, the quantity of fibre required can be maintained [32]. After very gradual introduction to the diet, to ensure appropriate microfloral population changes in the hindgut, straw can be used for this purpose.

On the other hand, in performance horses, athletic and behavioural performance can be a good indicator of whether their diet contains sufficient fibre and whether it also provides sufficient energy. Anecdotally, there is a reluctance to feed suitable amounts of fibre to performance horses (especially racehorses) because of concerns that gut fill and the gut fluid reservoir result in horses carrying additional weight. Research has shown that when high-quality, high-energy forages are fed, performance horses are able to be maintained on forage-only diets [33].

### 2.4. Recommended Fibre Intake for Horses

An extensive review of the recommended fibre intake for various classes of horses and ponies is provided by Harris et al. [8]. For full details on this subject, the authors direct readers there. In short, a lower limit of 1.5% of bodyweight in dry matter of conserved forage (equivalent to 15 g/kg of bodyweight (BW) or 7.5 kg/day for a 500 kg horse in dry matter) is recommended. On an as-fed basis for 90% dry matter hay, this is equivalent to 8.3 kg/day. Harris et al. [8] specify that 12.5 g/kg BW of dry matter is the absolute minimum recommendation (6.25 kg of dry matter per day or 6.9 kg on a 90% dry matter ‘as fed’ basis for a 500 kg horse) and that previously recommended amounts of 8–10 g of dry matter per kg BW are not acceptable to meet equine ethological needs and minimum health standards.

## 3. What Are the Behavioural Consequences of Feeding Less than the Minimum Daily Requirement of Fibre to Horses?

As with humans, who experience increased feelings of aggression and irritation when they report being hungry [34], associations between altered behaviour and feed limitations can also be found in horses. For example, horses on limited forage have been reported as high-risk for the emergence of oral stereotypies [35]. Stereotypies are behaviours that are repetitive and invariant without an obvious function or goal [36] and are not found in free-ranging horses. They are of significance because once they have been established as a mechanism for self-narcotization, they usually persist, a process known as emancipation [36]. It is not clear if these ethopathies, formerly known as ‘stable vices’, are caused by feelings of physical discomfort, hunger, or frustrated motivation to forage [37]. Unpacking the cause of unwanted behavioural responses can be difficult because they are often multifactorial in nature, with potential causes including diet, excitement, confinement, and frustration [14].

Nevertheless, there is clear evidence of behavioural consequences from feeding horses less than the recommended daily requirement of fibre because, even when the physiological need for nutrients is met, the motivation for foraging remains. The lack of opportunities to chew as a result of the way in which the diet is presented is known to be linked to not only oral stereotypies but also redirected behaviours, such as wood-chewing [38]. It is also possible that a lack of fibre may increase reactivity [39]. For the ridden horse, increased reactivity can increase the risk of rider falls. So, the importance of understanding these behavioural consequences is found not only in animal welfare but also in the safety of the horses’ handlers. This review will next discuss short- and long-term consequences to emphasise the need to prevent them.

### 3.1. Short-Term Consequences

Studies on free-ranging horses of different breeds have shown that, when their behaviour is not constrained, foraging is the major activity of a horse’s time budget. Horses will spend at least 50% of daylight hours foraging [14] (nighttime foraging was not included in this study) and up to 75% of daylight hours and 53% of the night [40]. The proportions of these time budgets help to explain the development of deleterious consequences for horses that endure an anthropogenic shift in their time budgets.

Crib-biting is a stereotypical behaviour presenting itself as the action of, with incisor teeth, seizing a fixed object and pulling back. The action is accompanied by a characteristic grunting noise, caused by the passage of air across the oropharynx. The behaviour is similar to windsucking, which is essentially crib-biting without the seizing of a fixed object [38]. Crib-biting is an abnormal behaviour [14] and has been suggested to be associated with diet and gastric ulceration [41]. There appears to be a relationship between peak crib-biting activity and fermentation in the caecum [42]. There is also evidence that both forms of the stereotypy are not initiated by mimicking other horses, in that control horses in studies did not copy crib-biting behaviour [42].

Wood-chewing is harder to define than crib-biting, as there is much more variation in the behaviour amongst different horses and environments and it is less repetitive. That said, it does appear that most crib-biting horses were previously wood-chewers [14]. Wood-chewing is sometimes considered to be related to dietary deficiencies [14]. However, in pasture-raised horses, normal foraging can include lignophagia (including bark- and wood-chewing) [43], which is thought to be especially frequent when the sward is low in fibre, such as in irrigated pastures [43]. Moreover, the behaviour can sometimes be reduced by increasing fibre content in the diet, suggesting the behaviour is induced by fibre deficiencies [38].

A study of foraging motivation fed horses two diets in different forms but with similar concentrations of crude protein and fibre [15]. The form of one diet was grass hay and the other was a concentrate pellet. Behavioural observations from a video record showed that, when fed hay, horses spent 61.5% of the observed time eating, compared to only 10% when fed pellets. Pellet-fed horses spent 11% of their time searching their stall, notably sifting through their wood-shaving bedding, in contrast to the hay-fed horses, who spent only 1% of their time engaged in searching behaviour [15]. It is unclear if the searching resulted in redirected grazing behaviour and the consumption of wood shavings. As the diets offered comparable nutrient levels, these differences in the horses’ time budgets suggest that a lack of time spent chewing may boost horses’ motivation to source fibre. Even though concentrated foods may be more rewarding [44], horses prefer substrates that take more time to consume and increase chewing rates by four times [15]. Hanis et al. [45] also found a negative association between fibre content in the diet and consumption of bedding material. A similar negative association was found between fibre content in the diet and coprophagia [45,46]. Consumption of bedding, wood chewing, and coprophagy (i.e., eating faeces) may all represent attempts to increase fibre intake and foraging time or both and are observed predominantly in horses fed concentrates rather than horses on fibrous diets [14]. In foals, coprophagia is considered normal (possibly as a means of populating the hindgut with suitable flora for the current substrates), whereas in adult horses, it can indicate micronutrient deficiency [38].

When not able to fill their time foraging, ponies have been reported to spend time licking salt [47]. Interestingly, the reported intensity of this redirected behaviour was higher than in ponies that were needing to lick the same substrate to replenish salt losses from sweating during exercise. As such, it could be that such licking is more of an oral occupation due to not being able to forage than a search for nutrients [38].

In a study of the effects of feeding management on meat-production horses’ welfare, Raspa, et al. [48,49] found a positive correlation between the forage content of diets and time spent feeding and drinking, activities that provide horses with the important nutrients water and fibre. In contrast, horses on a high-starch, no forage diet showed more non-nutritive behaviours such as locomotion, biting, standing, kicking, and stereotypical behaviour [48]. The authors proposed that the increased time spent standing alert and locomoting were signs of distress and therefore may indicate compromised animal welfare.

In a study of breeding mares, increasing the daily amount of hay offered increased positive social interaction and bonding [50]. In contrast, when forage was withdrawn, the mares showed antagonistic social interactions, such as aggression. Aligning with the findings of Raspa et al. [48], these mares were also observed to show more standing (alertness) and movement, suggesting higher levels of stress [50]. Conspecific aggression can compromise the safety of not only other horses in the herd but also the personnel handling them. Both Raspa et al. [48] and Benhajali et al. [50] found that reducing starch and increasing fibre in the diet significantly reduced intergroup aggression. With forage not available ad libitum, horses have been found to show aggression prior to feeding, especially when fed a concentrate meal [51].

Additionally, Fureix et al. [52] found a correlation between chronic or acute pain and aggression towards humans, especially when associated with human interactions (i.e., riding). As the physiological consequences of concentrate feeds that are low in fibre or high in starch can be very painful to the horse, they can potentially cause aggression towards humans [53].

In any social group of horses that has been denied ad libitum foraging opportunities, feeding activities can become competitive. This can increase the rate of consumption and, when the feed also does not contain sufficient fibre, there are potential physiological consequences. Rapid consumption often results in inadequate mastication. Fibre can provide the sensation of gut fill, reducing the need for ongoing consumption as the horse feels satisfied [9]. Fibre also requires chewing and the concomitant production of saliva that, once swallowed, lowers acidity in the stomach. Competitive behaviour and rapid consumption can be prevented by either providing fibre prior to feeding concentrates or increasing fibre content within concentrated rations [38].

### 3.2. Long-Term Consequences

If a horse’s diet does not meet the minimum daily fibre requirement of 1.5 kg per 100 kg of bodyweight, the sort of behavioural issues described above will emerge and persist. In addition, clinical issues may become apparent, one of them being Equine Gastric Ulcer Syndrome (EGUS). Although many horses with EGUS are asymptomatic (meaning that the condition often goes unnoticed/undiagnosed), the behavioural changes considered to be clinical signs of EGUS are poor performance, listlessness, depression, and poor appetite [54]. EGUS is considered a long-term consequence, as ulcers do not heal without a change in feeding practices. Moreover, without such changes, medication will only serve as a short-term solution, and new ulceration is to be expected without a change in feeding practices. Changing the diet and providing freedom of movement can prevent and cure ulcers, thus restoring more normal behaviour [54]. Pasture turnout minimises the risk of EGUS, with studies showing that even ad libitum forage availability for stabled horses does not prevent ulcers [55].

It is recognised that foal experiences in their early weeks and months of life can have long-term consequences on behaviour [56]. For example, prior to weaning, providing foals with a supplementary diet can moderate the negative experience of being separated from their dams [57]. This preferred supplementary diet should contain more fat and fibre than starch and sugar. This dietary composition is associated with decreased reactivity, not least to novel objects [38]. This dietary influence on reactivity indicates that a fat and fibre diet can reduce stress levels, increasing welfare for foals, especially when required to adapt to new challenges such as those that often accompany weaning [58].

It appears that foraging and the extended time needed to consume high-fibre diets underpin the behavioural impact of fibre in the diet. Consumption of fibre in concentrated forms, such as pellets, can also be slowed down with the use of foraging devices. These are devices aiming to prolong food-handling time [59]. As they slow down the rate of consumption, they can help to moderate the horse’s time budget so that it mimics those associated with normal foraging behaviour. Horses are known to paw and even bite at foraging devices, which has been interpreted as an expression of frustration [59]. However, even in the free-ranging state, horses occasionally need to paw to expose feed, for example, from underneath snow [23].

The impacts of various management and veterinary interventions on the welfare of horses are expected to vary with the invasiveness of the procedures and the prior histories of the horses that undergo them. An international panel assessed the impact of common practices on animal welfare using the Five Domains Model [60] to assess the welfare effects of nutrition, physical environment, health, and behavioural interactions on the horse’s inferred mental state. Using this model, interventions can be assessed as either enhancing or compromising welfare [61]. When considering diet, the expert panel established that while a no-forage diet has an important effect on nutrition, its primary impact is on behaviour.

Notably, the panel commented on the risk of frustration before delivery of forage, indicating the horses’ motivation for consumption of forage may equal or even surpass the motivation for concentrates (especially in horses that are fibre-deprived). They emphasised that horses value a choice of food and the freedom to locomote, underlining the importance of not only the content but also the distribution and form of dietary fibre. To sum up, in the context of diet, the panel recommended a varied pasture that included the opportunity to browse as the optimal environmental enrichment for confined horses [60].

## 4. What Are the Physiological Consequences of Feeding Less than the Minimum Daily Requirement of Fibre to Horses?

The horse’s gastrointestinal tract (GIT) is adapted to almost continuous grazing and the intake of dry matter high in fibre. Changing this behaviour or the form of diet will therefore affect the GIT. This can have serious health consequences [8].

As food enters the stomach from the oesophagus, it initially reaches the dorsal portion of the stomach, which has a similar lining and pH as the oesophagus. The pH differs across the stomach area. The dorsal area is neutral and comprises approximately one-third of the total stomach area. The other two-thirds are acidic (pH 3–6), and acidity increases with closer proximity to the pylorus (pH 1.5–3) [28]. Horses produce saliva chiefly during the action of chewing, in contrast to dogs that salivate in anticipation of a meal. This is another reason why the provision of long fibre is so important. Saliva has a buffer capacity for acidity in the stomach. Chewing increases saliva production. The continual grazing with only short periods of no feed intake is therefore important for providing the saliva buffer [28].

The stomach lining also varies between the two regions of the stomach. The dorsal part is the non-glandular squamous region, which is separated from the glandular region by the margo plicatus. The non-glandular region lacks the defence mechanisms in the mucosa to protect it from acidic juices found in the glandular region [62]. From the stomach, the digesta moves through the small intestine towards the hindgut, where fermentation takes place, as horses are hindgut fermenters [4]. In the small intestine, non-structural carbohydrates, protein, and dietary fat are hydrolyzed by enzymes [16]. A total of 72 lactic acid-producing bacteria isolates were found in the hindgut of horses, besides streptococci [17]. Starch-containing ingesta that escapes digestion in the small intestine greatly increases this lactate production, causing a drop in pH.

The following section discusses the main physiological consequences in the GIT of horses related to fibre and starch intake in two anatomical locations: the stomach and the hindgut.

### 4.1. Stomach

EGUS is a term that has been used since 1999 and it describes gastric ulcerations in horses. Since then, there has been a push to investigate EGUS in the different regions of the stomach separately (see Table 1). This resulted in the addition of more specific terminology, i.e., ESGD and Equine Glandular Gastric Disease (EGGD) [29].

Studies show contradicting results with regard to predisposing sex or age. However, there appears to be a greater risk for certain breeds, such as thoroughbreds, compared to cold-blooded breeds [29]. Little is known about the influence of diet on EGGD [29], therefore this section focuses on EGUS in general and ESGD. Most studies have focused on the prevalence of EGUS in the racing industry [63]. Other disciplines should not be neglected, as, for example, in endurance horses, prevalence has been found to be 67% [64]. This is similar to the prevalence under broodmares on pasture, at 70.9% [65], and slightly higher than in show horses, at 58% [66].

#### 4.1.1. Risk Factors

Risk factors vary and appear to intertwine, as not one stands out alone. They all cause the mucosa in the squamous region to be increasingly exposed to acid [29]. The severity of the ulcerations is directly related to the duration of exposure [67,68].

Exercise can cause the acidic gastric juices to move from the glandular region into the non-glandular region. This is caused by movement-induced intra-abdominal pressure, resulting in stomach compression. This can then cause the gastric juices to splash up against the squamous mucosa, resulting in randomly dispersed ulcerations [68]. This occurrence is aggravated on an empty stomach. Feeding forage, which can form a barrier between acidic juices and the squamous mucosa, within the 30 min before exercise will therefore limit splash ulcerations [69]. The severity of ulcerations is directly related to the duration of exercise [68]. Additionally, exercise can have the same effect as stress and increase serum gastrin concentrations [64].

Diets high in starch are often associated with being low in fibre. These high-starch, low-fibre diets put horses at greater risk of EGUS. Starch consumption increases the production of VFA [29,67] in the stomach and reduces buffering capacity due to its lower chewing requirement [62]. With less chewing, there is lower saliva production, which then reduces the buffering of gastric fluids [62]. Reducing time spent without forage will increase that buffer capacity and can subsequently reduce the risk of ESGD [29,69]. The VFA damages the outer squamous epithelial-cell barrier through penetration [69,70], making the mucosa vulnerable to ulcerations. Providing sufficient forage results in a greater reduction in risk than starch consumption, which increases risk. The addition of fibre to the starch diet can reduce acid motility, according to Hepburn [69], whereas this is contradicted by the findings of Chapa [71]. Changing horses with EGUS onto a diet free of starch that is fibre- and fat-enriched is highly effective for improving both ESGD and EGGD [72].

However, the fibre type is important, with lucerne hay being reported to be better than grass hay in resolving gastric ulceration. That said, while having a component of legume hay in a diet appears to be beneficial where EGUS is a concern, legume hay-only diets should not be recommended in practice.

The importance of water is stressed as water deprivation increases the risk of EGUS [69]. Stress can cause an increase in serum cortisol concentrations, which are mirrored by serum gastrin concentrations. Hydrochloric acid (HCl) secretion is indirectly stimulated by gastrin; therefore, stress can result in an increased production of HCl [69].

#### 4.1.2. Signs and Diagnosis

The signs of EGUS vary greatly among horses and appear to be specific to the horse’s individual differences (see Table 2). There has been no association found between the severity of the ulcerations and the severity of the clinical signs. The signs are often misinterpreted as non-EGUS-related behaviour [29,62]. In foals, signs can often be very subtle and are easily overlooked [67].

With the great variety of clinical signs among individual horses, special care should be taken when including these as a diagnostic tool. They appear to be too non-specific, and the association with EGUS is poor [29]. Clinical signs can therefore only result in suspicion in need of confirmation [69].

EGUS can be definitively identified only through gastroscopy [29]. ESGD and EGGD can occur independently of one another. The presence or absence of one does not provide any information on the occurrence of the other. Heweston et al. [73] investigated the usefulness of a blood sucrose test for EGUS diagnosis in foals. They found this to be a useful screening test prior to gastroscopy that gave a reliable indication as to whether gastroscopy was needed. It, however, lacks specificity and therefore cannot be used as a diagnostic tool. Shawaf, et al. [74] also investigated diagnostic options in the form of biomarkers and found no specific biomarkers. The diagnosis being limited to clinical signs and definitive only with a gastroscopy further emphasises the importance of preventing EGUS through feed management.

### 4.2. Hindgut

The hindgut, with its composition of bacteria, fungi, and protozoa, is an optimal environment for microbial fermentation at pH > 6.2. In certain situations, starch is not well digested in the small intestine of the horse. When grains containing largely indigestible starch, like corn or barley, that are not ‘cooked’ using some form of heat and moisture prior to feeding (e.g., via extrusion, micronizing, or boiling) are fed, or when individual horses have low levels of the starch digesting enzyme α-amylase, varying proportions of starch escape enzymatic digestion, resulting in fermentation in the hindgut, producing excess VFA and lactic acid [17]. Therefore, the addition of starch to the diet that escapes digestion in the small intestine has the capacity to lower pH in the hindgut, potentially causing hindgut acidosis, which can subsequently lead to laminitis and colic [4,20]. Gut motility is also influenced [69]. Furthermore, there is major disruption to the hindgut microbiome with far-reaching effects on animal health, behaviour, and welfare [75].

Colic can be caused by dehydration in the colon, associated with high-starch diets contrary to high-fibre diets, causing an intestinal obstruction [4]. Another cause of colic is the consumption of indigestible content, such as sand [76]. This can be an unintended intake caused by foraging-replacing behaviour when the diet lacks forage and fibre. Hay consumption can be a cause of colic, specifically hay produced from fine grasses. This is potentially caused by limited chewing, reducing saliva production, and resulting in the dry compaction of fine fibres [76]. This indicates the great importance of saliva production, not only for its buffer capacity but also to provide fluid to prevent the compaction of digesta.

Laminitis is caused by hindgut acidosis caused by the proliferation of lactic acid-producing bacteria, leading to a reduction in hindgut pH [77]. Lowered pH caused by hindgut acidosis causes lamellar inflammation, which subsequently can result in laminitis [4]. Horses can show signs of lameness within 24 h after ingestion of the starch-containing feed that caused lactic acid levels to rise in the hindgut [17]. Laminitis will not occur in every animal, as some horses are susceptible whereas others are not. Prevalence is greater in ponies than in horses [78]. Another cause for laminitis is found in non-structural carbohydrates, found in pasture or hay. Therefore, the fibre source is extremely important in laminitis prevention [77], and restriction of pasture access can be required, which is often confused with a need for restriction of fibre [78].

High-fibre diets are key to keeping hindgut microbial communities stable and preventing metabolic disorders. The increased energy supplied by high-starch diets can also be provided in a fibrous form, such as sugar-beet pulp [4,79]. For the diet, the form and quality of fibrous feed have no significant influence on the bacteria, fungi, and protozoa concentrations in the hindgut, whereas a small addition of starch can drastically change these concentrations [21]. This further emphasises the importance of providing high-energy protein in a fibrous form rather than a starch-containing diet.

## 5. Putative Justifications for Feeding Less than the Minimum Daily Requirement of Fibre to Horses

The volume of studies stipulating fibre requirements for horses is a good indication of its importance. Even though there is sufficient evidence available that a low-fibre, high-starch diet has harmful physiological consequences and subsequently can result in welfare issues [8,49,80], it seems that this information does not reach the entire horse-owning community. It is also unclear whether the evidence provided by these studies has resulted in horses generally receiving adequate forage because most articles conclude with dietary recommendations but rarely audit whether these recommendations are met. A study in the Netherlands showed that a third of horse owners consider 5 kg of fibre sufficient (combined with 5 kg of high starch concentrates) [3], and the average amount of forage fed in the Turkish race industry was 1% (±0.4) DM of BW per day [79]. It will be difficult to find a singular justification for feeding less than the minimum daily requirement of fibre. This largely reflects the diversity of people who are horse owners, ranging from breeders, professionals, and recreational riders to people owning horses for companionship and for profit [81]. These owners all obtain information from a variety of sources [1,2]. Nevertheless, with all the evidence available, the justifications people offer for not feeding horses the minimum daily requirement of fibre merit scrutiny.

### 5.1. Lack of Knowledge

Hoffman et al. [2] reported a lack of general knowledge regarding horse nutrition among horse owners in New England, USA. Specifically, even among veterinarians, there are often gaps in knowledge about how to assess forage quality, measure the amount to feed, and calculate the horse’s nutrient requirements. Many of the participants in the study had consulted veterinarians for advice on feed management for their horse, but other common sources of information were trainers and the internet. From the latter two sources, it is unclear whether the information sourced had any scientific basis. Nutritionists were consulted less than feedstore personnel and magazines, and only slightly more often than friends. This indicates that horse owners rarely consult professionals regarding their nutritional questions, and faulty advice can be spread easily and rapidly, further increasing the presence of knowledge gaps. Not knowing the fibre content of the feed makes it difficult to assess if one is meeting one’s horse’s requirements. Not knowing how to measure the feed can regularly result in not feeding the amount intended [82]. In the Netherlands, the main source of information is fellow horse enthusiasts, followed by veterinarians and farriers. Almost half of the respondents in the study by Visser and Van Wijk-Jansen [3] said they regarded their horse almost as a partner or child, which indicates they would have their best interests at heart. However, this did not necessarily manifest in the best feeding practices, with many owners instead prioritising concentrate feed over forages, which may indicate horse owners are subconsciously making feeding decisions based on what they would prefer to eat as opposed to making decisions based on what is in the best health interests of their horse.

### 5.2. Confusing Fibre with Forage

Often, when thinking of dietary fibre for horses, only forages are considered. This is not surprising, as the words are often used interchangeably, without any mention of other sources of fibre appearing in the information available in books and on websites [83,84]. A simple Google search of ‘What to feed my horse’ shows that the top results [85,86], all focused on animal welfare, but none mentioned fibre intake from any source other than forages. Forages are still often considered a roughage source used to provide bulk rather than nutritional value [87]. This does not only tie in with it being considered dead weight, which can slow the horse down [84], but also with horse owners replacing the forage with concentrate feeds in an ill-advised attempt to do what is best for their horse [3].

### 5.3. Tradition

People tend not to follow detailed instructions with a reliable level of accuracy, which could reflect a lack of confidence in the information they contain. Kaya-Karasu, et al. [88] found that racehorse trainers in Turkey who fed commercial mixed feeds were feeding less than the recommendation, even though the racehorses being fed would have a high energy requirement. They also found that often nutritionist advice was slightly altered or diluted, and feed was not balanced to meet requirements for racehorses according to the National Research Council [89]. In particular, starch was fed at levels well above those recommended. The justification for nutrition-related decisions was that they were based on tradition.

Richards, Hinch and Rowe [1] asked trainers about their decisions to include grain in the diet but did not report on how such decisions affected subsequent levels of hindgut fermentation. They report that nutritionists were involved in only 5.6% of the decisions and that 6.9% of the decisions were based on tradition. Only 1.4% of decisions were based on research and the most recent data. It also seems logical that the expense or availability of forage and the efforts in handling and storing large bales of forage may deter some owners from meeting their horses’ fibre requirements.

### 5.4. Reduced Performance

Concerns regarding performance being compromised by horses carrying extra mass (dead weight) in their GIT from particularly forage or pasture intake are expected in the racing industry. For example, Williamson, Rogers and Firth [80] propose trainers often justify offering only limited access to pasture by a perceived need to minimise gut fill. The association between hay consumption and water intake is also thought to be compromising performance, and there is also a fear that gut fill from forage could result in reduced concentrate intake, which reduces digestible energy available for racing [90]. Increased exercise appears to have a negative effect on feed intake, to the extent that it falls short of the horses’ energy requirements [91]. Feeding less forage and increasing energy intake per bite could be motivated by attempts to maintain the horse’s energy intake. Stable air hygiene may deter the feeding of certain forages associated with bronchospasm [92]. Inflammation of the airways as a result of dust or other respiratory irritants is associated with decreased performance in stabled racehorses [93]. Olave, et al. [94] found that dry hay increases the risk of inflammation of the horses’ airways, which can be greatly reduced by using another fibre source, such as haylage [95]. Decisions in this domain may represent a trade-off between the welfare of the horse and the chances of winning. McLean and McGreevy [95,96] discuss the issue of competitiveness. It can result in a trade-off between the welfare of the horse and the chances of winning. They suggest that many a rider, trainer, or owner might at some point deliberately choose winning over doing the right thing by the horse.

## 6. Opportunities for Future Research

There is a multitude of research articles that confirm the damage that can result from not feeding horses their fibre requirement and from feeding a high starch content. Many of these reports conclude with recommendations to improve feeding management. There would be scientific value in returning to these specific trainers and horse owners to see if these recommendations were implemented and what justifications were offered if the recommendations were ignored. A general survey of horse owners and trainers to investigate current feeding practices and the psychology behind decisions about horse nutrition could reveal opportunities to implement optimal feeding practices. Finding ways to make feeding information more uniform and accessible to horse owners has the potential to greatly increase horse welfare through optimised feeding.

## 7. Conclusions

After water, fibre is the most important component of a horse’s diet. Its value in providing energy is too often underestimated. Failure to meet the fibre requirement of 1.5% of the horse’s bodyweight and the opportunity for foraging for a minimum of eight hours a day (not going without this opportunity longer than five hours) can have both physiological and behavioural consequences. Providing hay can reduce crib-biting, wood-chewing, coprophagia, the consumption of bedding, aggression, and stress, and subsequently increase social bonding and affiliation with conspecifics. Providing insufficient fibre can compromise welfare and increase intragroup aggression. More research into the effect of fibre intake specifically on behaviour, whether supplied as forage or concentrate (as in sugar-beet pulp, for example), would be beneficial.

To provide an energy source for horses, rations often include starch rather than fibre. This can result in health issues related to GIT in horses. Choosing a fibrous alternative for starch in a high-energy diet will greatly reduce the risk of EGUS and acidosis and improve digestion, GIT pH, body condition, behaviour, and performance. In many cases, failing to feed the horse its fibre requirement reflects a lack of knowledge. Other attempted justifications are tradition and prioritising winning over welfare.

## Figures and Tables

**Table 1:** Terminology derived from EGUS and their descriptions [29].

EGUS	ESGD	Primary ESGD	ESGD in horses with otherwise intact GIT, assessed via a grading system.
		Secondary ESGD	EGGD occurring secondary to delayed gastric emptying as a result of another pathology.
	EGGD	Anatomically	Described in relation to region: cardia/fundus/antrum/pylorus.
		Descriptively	Described in relation to severity (mild/moderate/severe), mucosal contour (flat/raised/depressed ± bloodclot), or epithelial appearance (hyperaemic/haemorrhagic/fibrinosuppurative).

**Table 2:** Overview of clinical signs and manifestations of EGUS.

Clinical Sign	Expressed As	Source
Reduced appetite	Reduced overall feed intake.	[69]
Changes in feed intake	Reduced feed intake of certain ingredients, such as grain or forage.	[62]
Weight loss	Resulting from reduced appetite or an increased metabolic rate caused by chronic mild pain.	[62,69]
Poor coat quality	Dull and rough-haired coat, which could be related to chronic pain or weight loss.	[62,69]
Reduced performance	Behavioural changes and lack of focus.Decreased oxygen consumption.	[62,69]
Behavioural changes	Changes in attitude towards handlers or other horses. Often expressed as increased fear and flight responses or, in contrast, abnormally quiet or dull	[62,69]
Abdominal pain	Various, potentially sick horses have been found in the recumbent position regularly, with increased urinating in a stretched-out position and discomfort from tightening the girth.	[62,69]
Colic	A mild to moderate form of colic that is recurrent.	[62,69]
Silent	Non-clinical occurrence of EGUS or subclinical occurrence where there are no clinical signs observed, however improvement in behaviour was observed after treatment.	[29,67]

## Data Availability

Not applicable.
